# Synthetic, non-intoxicating 8,9-dihydrocannabidiol for the mitigation of seizures

**DOI:** 10.1038/s41598-019-44056-y

**Published:** 2019-05-23

**Authors:** Mark Mascal, Nema Hafezi, Deping Wang, Yuhan Hu, Gessica Serra, Mark L. Dallas, Jeremy P. E. Spencer

**Affiliations:** 1 Department of Chemistry, University of California Davis, 95616 Davis, CA USA; 2 School of Chemistry, Food and Nutritional Sciences, and Pharmacy, University of Reading, Whiteknights, Reading, RG6 6AP Berkshire, UK

**Keywords:** Molecular neuroscience, Pharmacodynamics

## Abstract

There can be a fine line between therapeutic intervention and substance abuse, and this point is clearly exemplified in herbal cannabis and its products. Therapies involving cannabis have been the treatment of last resort for some cases of refractory epilepsy, and this has been among the strongest medical justifications for legalization of marijuana. In order to circumvent the narcotic effects of Δ^9^-tetrahydrocannabinol (THC), many studies have concentrated on its less intoxicating isomer cannabidiol (CBD). However, CBD, like all natural cannabinoids, is a controlled substance in most countries, and its conversion into THC can be easily performed using common chemicals. We describe here the anticonvulsant properties of 8,9-dihydrocannibidiol (H2CBD), a fully synthetic analogue of CBD that is prepared from inexpensive, non-cannabis derived precursors. H2CBD was found to have effectiveness comparable to CBD both for decreasing the number and reducing the severity of pentylenetetrazole-induced seizures in rats. Finally, H2CBD cannot be converted by any reasonable synthetic route into THC, and thus has the potential to act as a safe, noncontroversial drug for seizure mitigation.

## Introduction

**Figure 1:**
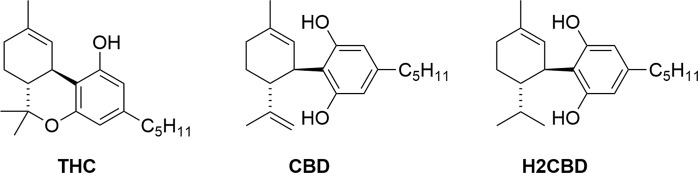
Structures of the natural cannabinoids and synthetic H2CBD.

There is currently a great deal of research activity around the potential for phytocannabinoids, *i*.*e*. compounds that occur naturally in the hemp plant (*Cannabis sativa*), to treat a wide range of medical conditions, including anxiety, glaucoma, epilepsy, spasticity, inflammation, neurodegenerative diseases, affective disorders, and even cancer^1,2^. Indeed, a Medline database search using the term “cannabis” returns >14,000 journal hits since the year 2000, including nearly 900 literature reviews with cannabis- or a cannabinoid-related term in the title. The opportunities around the therapeutic potential of cannabinoids are however weighed against a range of drawbacks, including adverse health effects, potential for abuse, cognitive and motor impairment, psychiatric disturbances, legal issues, and the environmental impacts of marijuana cultivation^3–5^. Beyond this, herbal cannabis has been shown to contain >500 chemical entities, including around 100 cannabinoids alongside a variety of other terpenes, phenolics, flavonoids, lipids, and steroids, the toxicity and mutagenic nature of which are largely unexplored^6,7^. Of the two major cannabinoids that occur in cannabis, *i*.*e*. ∆^9^-tetrahydrocannabinol (THC) and cannabidiol (CBD) (Fig. 1), the deleterious effects (intoxication, ataxia, tachycardia, somnolence, dry mouth, and hyperphagia) are primarily attributed to the former, and for that reason CBD has often been singled out for pharmacological investigations^8^.

While the use of CBD appears to circumvent most of the drawbacks of using cannabis preparations, there remain significant issues associated with its use: (1) All marijuana extracts, including CBD, are controlled substances in most countries, although some have decriminalized the use of cannabis primarily for therapeutic purposes. (2) CBD is derived by extraction from the cannabis plant. A wide range of impurities may be present, and there is a growing concern for contamination by pesticides^9^, particularly in the current, largely unregulated climate. (3) Even if pure CBD is marketed, the deliberate chemical conversion of CBD to THC is technically trivial^10,11^. Were CBD to become freely available, it could lead to a culture similar to that of the pseudoephedrine-to-methamphetamine “meth lab” phenomenon, except that conversion of CBD to THC would involve a logistically far simpler chemical transformation. Pure THC, containing no CBD to antagonize its psychotropic effects^12^, is a potentially dangerous drug. (4) A collateral liability of the derivation of CBD from cannabis is the cultivation of hemp, with potential environmental impacts in terms of heavy water usage and pesticide/herbicide effluent burden. Legalization of marijuana will inevitably also lead to private cultivation using methods not intended to manage potential environmental damage. (5) Finally, the impact of legalized cannabis on healthcare systems, which in the US has been recently highlighted in the areas of accidental injuries^13^, unintentional ingestion of cannabis edibles by children^14^, and reproductive health^15,16^, may be considerable.

Among the potential medical indications of cannabis, it can be argued that its highest profile use is as an antiepileptic. Epilepsy is the general term given to a spectrum of conditions characterized by recurrent, unpredictable seizures, the consequences of which often have a profound effect on quality of life. Historical and anecdotal evidence, along with a number of case studies documenting the practically unique efficacy of cannabis to treat some refractory cases of epilepsy^17,18^, have led to strong advocacy in favor of the legalization of marijuana^19^. This has recently culminated in US Food and Drug Administration (FDA) approval of Epidiolex (purified herbal CBD) for the treatment of Lennox-Gastaut and Dravet syndromes. Preclinical evidence for anticonvulsant activity of CBD and THC in acute animal models of seizures is also strong^20,21^.

Here, we describe the antiepileptic potential of 8,9-dihydrocannabidiol (H2CBD), a synthetic cannabinoid that differs structurally from CBD only by the saturation of the exocyclic carbon-carbon double bond. An immediate advantage of H2CBD is that, despite its similarity to CBD, it is not present in cannabis extracts and therefore not presently a controlled substance. Importantly, there is no reasonable synthetic route for the conversion of H2CBD to THC, in stark contrast to CBD itself. Although H2CBD has been prepared from natural CBD^22^, we opted to employ an efficient, fully synthetic approach in order to avoid the intermediacy of any scheduled substance and thereby also circumvent any necessity for the cultivation of hemp to supply H2CBD.

H2CBD has previously been the subject of a limited number of studies involving cannabinoid pharmacology. Consistent with CBD, H2CBD shows (1) an inhibitory effect on cytochrome P450^23,24^, which can be measured by CO complex formation during hepatic microsomal metabolism of H2CBD^25,26^, and (2) antioxidant activity quantified by inhibition of the production of reactive oxygen intermediates, nitric oxide, and tumor necrosis factor in murine macrophages^27^. While there is evidence to show that the documented sedative effects of CBD^28,29^ may be due to *in vivo* conversion to THC in the acidic gastric environment^30,31^, H2CBD, which cannot undergo this reaction, shows little if any evidence of narcotic activity^32^.

Mechanistically, synaptic transmission can be regulated by activation of the cannabinoid receptor CB1, and endocannabinoids are known to play a protective role in central nervous system disorders, particularly those associated with neuronal hyperexcitability^33^. Herbal cannabinoids are CB1 agonists and have been shown to exhibit CB1 receptor-dependent anticonvulsant activity in models of epilepsy^34^, while conversely, the application of the CB1 receptor antagonists induces epileptiform activity in these models^35^. However, the precise role of CB1 activation in seizure mitigation has yet to be fully elucidated, and the protein targets of CBD (and by analogy, H2CBD) have not all been identified, so we do not at this point propose a mechanistic interpretation of the action of H2CBD in this context.

## Results and Discussion

**Figure 2:**
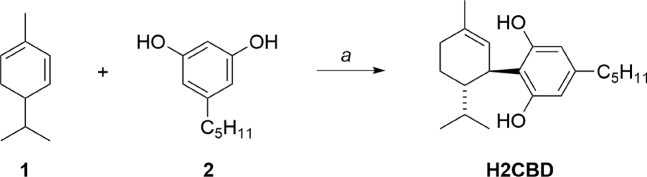
Preparation of synthetic H2CBD. *Reagents and conditions*: *a*. p-toluenesulfonic acid monohydrate (0.3 eq), benzene, RT, 1 h.

A simple, one-step synthetic approach to H2CBD could be based on the work of Crombie and co-workers^36^. Optimization of this reaction led to the isolation of H2CBD in a 71% yield (Fig. 2). The route is economically practical at scale: α-Phellandrene **1** is an inexpensive natural fragrance chemical^37^, and olivetol **2** is also commercially available but can be prepared in bulk using a two-step method starting from the flavor ingredient 3-nonen-2-one^38^.

In the seizure mitigation study, a total of 60 male Wistar Han rats were randomly divided into 5 groups of 12 animals each and received either vehicle (ethanol, Cremophor EL, 0.9% w/v saline; 2:1:17), vehicle plus a positive control (CBD; 200 mg kg^−1^), or vehicle plus H2CBD (50, 100, or 200 mg kg^−1^) via intraperitoneal injection one hour prior to administration of the convulsant agent pentylenetetrazole (PTZ; 85 mg kg^−1^ in 0.9% w/v saline). Seizure activity was monitored as described in the Methods Section.

**Figure 3:**
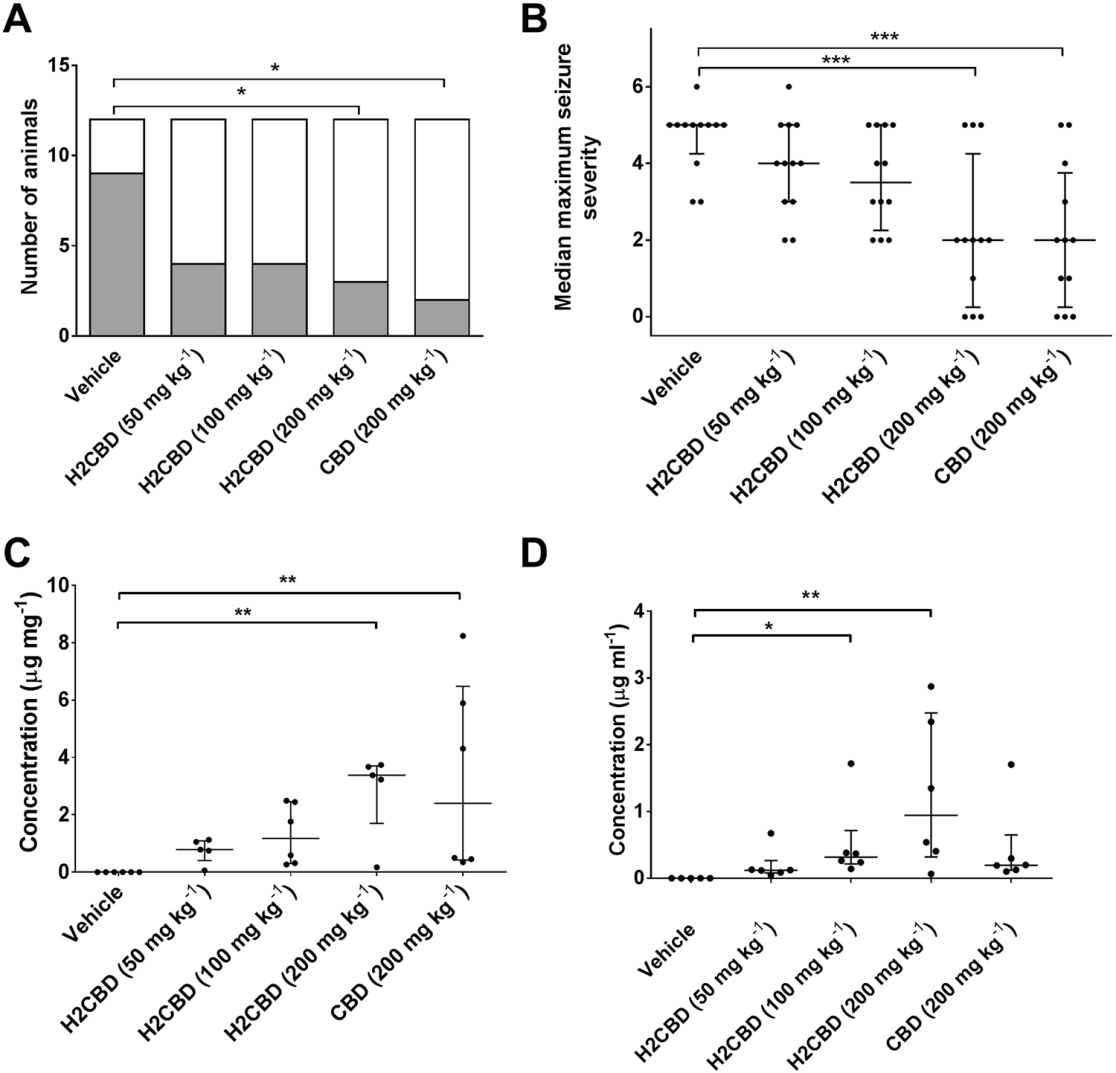
Effect of H2CBD upon acute, PTZ-induced primary generalised seizures in rat. Effects of vehicle, H2CBD (50, 100 200 mg kg^−1^), and CBD (200 mg kg^−1^) treatments upon (**A**) the proportion of animals exhibiting tonic-clonic seizures (gray shaded area) and (**B**) median (middle bars), interquartile range (upper and lower bars) and individual (•) maximum seizure severity, following PTZ administration. *P < 0.05; ***P < 0.001. Error bars in (**B**) show SEM. n = 12 animals per group in each case. (**C**) Brain and (**D**) blood concentrations of H2CBD (50, 100 200 mg kg^−1^) and CBD (200 mg kg^−1^) assessed via post-mortem samples obtained 90 minutes after cannabinoid administration. n ≥ 5 animals per group. Plots show median (middle bars), interquartile range (upper and lower bars) and individual animal (•) results. *P < 0.05; **P < 0.01.

An overall effect of treatment upon the percentage of animals that exhibited tonic-clonic seizures was found (χ^2^ (4) = 10.48; P = 0.033), where pairwise comparisons revealed that significantly fewer animals that received CBD (200 mg kg^−1^) or H2CBD (200 mg kg^−1^) exhibited tonic-clonic seizures than the vehicle treated group (P < 0.05 in both cases) (Fig. 3). Furthermore, maximum seizure severity was also affected by treatment (H = 18.96; P < 0.001), where pairwise comparisons revealed that animals that received CBD (200 mg kg^−1^) or H2CBD (200 mg kg^−1^) exhibited significantly less severe seizures as coded by the Racine scale than the vehicle treated group (vehicle median: 5 (4.25–5 IQR), H2CBD 200 mg kg^−1^: 2 (0.25–4.25 IQR), CBD 200 mg kg^−1^: 2 (0.25–3.75 IQR), P < 0.001 in both cases).

Analysis of blood and brain tissue obtained from animals in each treatment group post-mortem revealed an overall effect of dosing upon blood (H = 17.00; P = 0.0019) and brain tissue (H = 15.76; P = 0.0034) arising from detection of significant concentrations of H2CBD (100 mg kg^−1^: P < 0.05 (blood); 200 mg kg^−1^: P < 0.01 (blood); P < 0.01 (brain)) and CBD (200 mg kg^−1^: P < 0.01 (brain)) when compared with the vehicle treated group (Fig. 3).

These results unequivocally demonstrate that H2CBD exhibits a dose-dependent anticonvulsant action in acute, PTZ-induced generalised seizures in rats, with a maximal protective effect comparable to a matching dose of the established anticonvulsant CBD^39^. While these preliminary data provide a clear indication for the use of H2CBD as an anticonvulsant agent, further work will establish the inherent pharmacokinetic profile of H2CBD which, for the purposes of this study, was assumed to be identical to CBD, although indications from the bioanalyte results suggest differences in plasma and brain concentration at matching doses (200 mg kg^−1^), despite a comparable anticonvulsant effect. This may suggest the magnitude of the anticonvulsant effect of H2CBD could be being attenuated by suboptimal dosing intervals, preventing the effect from being assessed at maximal drug concentration.

In conclusion, it has been demonstrated that prophylactic administration of H2CBD (200 mg kg^−1^) to rats significantly reduces incidence of tonic-clonic seizures as well as maximum seizure severity as compared to vehicle treatment, with a comparable anticonvulsant effect as seen in positive control CBD. We summarize the advantages of H2CBD over CBD as a potential antiepileptic drug as follows: (1) Being fully synthetic, H2CBD is not a controlled substance and thereby may circumvent legal issues surrounding cannabis-based therapies. (2) The preparative approach to H2CBD is efficient, inexpensive, and scalable. Unlike CBD, which has to be isolated from a mixture of hundreds of other extractives and may also be contaminated with pesticides, synthetic H2CBD is easy to obtain in pure form. (3) Preparation of H2CBD from readily available, non-cannabis based precursors eliminates the necessity to cultivate marijuana and its attendant social and environmental concerns. (4) The comparable antiepileptic activity between natural CBD and synthetic, racemic H2CBD suggest that both enantiomers of H2CBD have comparable potency, consistent with literature studies on unnatural (+)-CBD^40^. (5) In contrast to CBD, there is no pathway from H2CBD to THC, either *in vivo* or in the laboratory. Thus, assuming the principal medical justification for pursuing cannabis-based therapies is their extraordinary anticonvulsant activity, and that all other indications (anxiety, chronic pain, nausea, anorexia, etc) can be effectively managed with non-controversial drugs^41^, H2CBD may find application as a safer alternative in terms of its lack of abuse liability and absence of psychotropic effects.

## Methods

### H2CBD preparation

Chemicals were purchased from Sigma-Aldrich and used as received. Based on a procedure reported by Crombie and coworkers^36^, a solution of olivetol (1.72 g, 9.54 mmol) and food-grade α-phellandrene (1.41 g, 10.4 mmol, 1.09 eq) in benzene (5 mL) was treated with p-toluenesulfonic acid monohydrate (0.545 g, 2.87 mmol) and the mixture was allowed to stir at room temperature for 1 h. The solvent was removed in vacuo and the residue was purified by silica gel chromatography using a gradient elution (100% hexanes to 10% diethyl ether in hexanes) to give H2CBD (2.14 g, 71%) as a dark yellow oil. Spectroscopic data (^1^H-NMR, ^13^C-NMR) were in full agreement with the literature.

### Antiseizure study

#### Animals

Male, Wistar Han rats (70–110 g; Harlan, Bicester, UK) were housed on a 12 h light–dark cycle, with food and water available *ad libitum*. All experiments were conducted in accordance with the UK Animals Scientific Procedures Act 1986 under a UK Home Office license and ARRIVE guidelines for reporting experiments involving animals^42,43^. A total of 60 rats were used.

#### Drug administration

Animals were randomly divided into 5 groups of 12 animals per group and received either vehicle (ethanol, Cremophor EL and saline (0.9%w/v NaCl), 2:1:17), a positive control (CBD; 200 mg kg^−1^; Sigma-Aldrich UK) or 8,9-dihydrocannabidiol (H2CBD; 50, 100 or 200 mg kg^−1^) via intraperitoneal injection 1 h prior to administration of the convulsant agent pentylenetetrazole (PTZ) to achieve brain cannabinoid T_max_. PTZ (85 mg kg^−1^ in 0.9%w/v NaCl) was administered intraperitoneally 1 h after drug or vehicle treatment. Seizure activity was video recorded for 30 min and video records blinded before offline review and coding using a modified Racine scale (0, normal behaviour; 0.5, abnormal behaviour; 1, isolated myoclonic jerk; 2, atypical clonic seizure; 3, bilateral forelimb clonus; 3.5, bilateral forelimb clonus with body twist; 4, tonic–clonic seizure with suppressed tonic phase; 5, fully developed tonic–clonic seizure).

### Analysis of plasma and brain samples

4,4-Dichlorodiphenyltrichloroethane (DDT, CAS: 50-29-3) was used as the internal analytical standard (IS). HPLC grade n-hexane, acetonitrile, water and ascorbic acid were purchased from Sigma Aldrich UK and Fisher Scientific. Stock standard solutions of CBD, H2CBD and DDT were prepared in acetonitrile (5 mg ml^−1^ and 1 mg mL^−1^) and stored at −20 °C until use. These were further diluted in acetonitrile:water (62:38), to achieve calibration concentrations of 0.1, 0.2, 0.5, 1, 5, 10 μg mL^−1^. Plasma samples were prepared for HPLC using a previously validated method^44^. Briefly, DDT (50 μg mL^−1^) was added to 150 μL of rat plasma sample as internal standard and plasma proteins were precipitated by the addition of ice cold acetonitrile (600 μL) followed by water (600 μL), with 1 min vortexing between additions. n-Hexane (3 mL) was added to each tube and following a 5 min vortex, tubes were centrifuged at 1160 × g for 15 min at 10 °C and the upper organic layer was carefully decanted by glass pipette and retained. The organic layer was evaporated to dryness under a stream of nitrogen at room temperature and reconstituted in 150 μL of the mixture of acetonitrile and water (62:38) prior to HPLC analysis.

For post-mortem brain analysis, brains were weighed and 1.5 x ice-cold solvent (90% acetonitrile; 10% water; 0.1% ascorbic acid) (w/v) was added followed by homogenization for 1 min. DDT (50 μg mL^−1^) was added to each homogenized brain tissue as internal standard, samples were mixed and allowed to equilibrate overnight at −20 °C. Samples were then centrifuged at 3500 rpm for 15 min and the top layer retained. Samples were dried by SpeedVac concentrator at room temperature (Savant SPD131DDA, ThermoFisher Scientific, UK) and reconstituted in 150 μL of the mixture of acetonitrile and water (62:38) for HPLC analysis.

An Agilent 1200 series HPLC (Hewlett–Packard, Palo Alto, CA, USA) equipped with a photodiode array detector was used for analysis. 30 μl of all samples were injected and separation was achieved using an ACE C18-PFP 150 mm 4.6 mm, 3 μm particle size column (Hichrom Ltd., Reading, UK), protected by an ACE C18-PFP 3 μm guard cartridge. The mobile phase was a mixture of acetonitrile and water in a ratio of 62:38 (v/v). The flow rate was set at 1 mL min^−1^ and the column temperature was maintained at 55 °C. The absorbance of the compounds of interest (CBD and H2CBD) was monitored at 220 nm.

Statistical procedures were performed using GraphPad Prism 7 (GraphPad Software, Inc., San Diego, CA, USA). A D’Agostino and Pearson normality test revealed that data describing maximum seizure severity and bioanalyte concentrations were not normally distributed. Therefore, assessment of differences within groups of these data types were assessed by a Kruskal–Wallis test with post-hoc Dunn’s tests. Drug effects upon the percentage of animals exhibiting tonic-clonic seizures were assessed by a chi-squared test with post-hoc Fisher exact tests.

## References

[1] Robson PJ (2014). Therapeutic potential of cannabinoid medicines. Drug. Test. Anal..

[2] Hill KP (2015). Medical marijuana for treatment of chronic pain and other medical and psychiatric problems: A clinical review. JAMA.

[3] Volkow ND, Baler RD, Compton WM, Weiss SRB (2014). Adverse health effects of marijuana use. N. Engl. J. Med..

[4] Hall W, Degenhardt L (2014). The adverse health effects of chronic cannabis use. Drug. Test. Anal..

[5] Ashworth K, Vizuete W (2017). High time to assess the environmental impacts of cannabis cultivation. Environ. Sci. Technol..

[6] Giese MW, Lewis MA, Giese L, Smith KM (2015). Development and validation of a reliable and robust method for the analysis of cannabinoids and terpenes in cannabis. J. AOAC Int..

[7] Cascio, M. & Pertwee, R. *Handbook of Cannabis* (Oxford, UK, 2014).

[8] Pisanti S (2017). Cannabidiol: State of the art and new challenges for therapeutic applications. Pharmacol. Ther..

[9] Subritzky T, Pettigrew S, Lenton S (2017). Into the void: Regulating pesticide use in Colorado’s commercial cannabis markets. Int. J. Drug Policy.

[10] Adams R, Pease DC, Cain CK, Clark JH (1940). Structure of cannabidiol. vi. isomerization of cannabidiol to tetrahydrocannabinol, a physiologically active product. conversion of cannabidiol to cannabinol. J. Am. Chem. Soc..

[11] Adams R, Cain CK, McPhee WD, Wearn RB (1941). Structure of cannabidiol. xii. isomerization to tetrahydrocannabinols. J. Am. Chem. Soc..

[12] Klein C (2011). Cannabidiol potentiates delta(9)-tetrahydrocannabinol (thc) behavioural effects and alters thc pharmacokinetics during acute and chronic treatment in adolescent rats. Psychopharmacol. (Berl).

[13] Li MC (2012). Marijuana use and motor vehicle crashes. Epidemiol. Rev..

[14] Wang GS, Roosevelt G, Heard K (2013). Pediatric marijuana exposures in a medical marijuana state. JAMA Pediatr..

[15] Gundersen TD (2015). Association between use of marijuana and male reproductive hormones and semen quality: A study among 1,215 healthy young men. Am. J. Epidemiol..

[16] du Plessis SS, Agarwal A, Syriac A (2015). Marijuana, phytocannabinoids, the endocannabinoid system, and male fertility. J. Assist. Reprod. Genet..

[17] Reddy DS, Golub VM (2016). The pharmacological basis of cannabis therapy for epilepsy. J. Pharmacol. Exp. Ther..

[18] Devinsky O (2016). Cannabidiol in patients with treatment-resistant epilepsy: an open-label interventional trial. Lancet Neurol..

[19] Maa E, Figi P (2014). The case for medical marijuana in epilepsy. Epilepsia.

[20] Hill, A. J., Hill, T. D. & Whalley, B. J. *The Development of Cannabinoid Based Therapies for Epilepsy*, 164–204 (2014).

[21] Rosenberg EC, Patra PH, Whalley BJ (2017). Therapeutic effects of cannabinoids in animal models of seizures, epilepsy, epileptogenesis, and epilepsy-related neuroprotection. Epilepsy Behav..

[22] Gaoni Y, Mechoulam R (1968). The iso-tetrahydrocannabinols. Isr. J. Chem..

[23] Jiang R, Yamaori S, Okamoto Y, Yamamoto I, Watanabe K (2013). Cannabidiol is a potent inhibitor of the catalytic activity of cytochrome p450 2c19. Drug Metab. Pharmacokinet..

[24] Bornheim LM, Everhart ET, Li J, Correia MA (1993). Characterization of cannabidiol-mediated cytochrome p450 inactivation. Biochem. Pharmacol..

[25] Watanabe K, Narimatsu S, Gohda H, Yamamoto I, Yoshimura H (1988). Formation of similar species to carbon monoxide during hepatic microsomal metabolism of cannabidiol on the basis of spectral interaction with cytochrome p-450. Biochem. Pharmacol..

[26] Usami N, Tateoka Y, Watanabe K, Yamamoto I, Yoshimura H (1995). Formation of carbon monoxide during mouse hepatic microsomal oxidative metabolism of cannabidiol; identification and determination. Biol. Pharm. Bull..

[27] Ben-Shabat S, Hanus LO, Katzavian G, Gallily R (2006). New cannabidiol derivatives: Synthesis, binding to cannabinoid receptor, and evaluation of their antiinflammatory activity. J. Med. Chem..

[28] R de Mello Schier A (2014). Antidepressant-like and anxiolytic-like effects of cannabidiol: a chemical compound of cannabis sativa. CNS Neurol. Disord. Targets (Formerly Curr. Drug Targets-CNS Neurol. Disord..

[29] Blessing EM, Steenkamp MM, Manzanares J, Marmar CR (2015). Cannabidiol as a potential treatment for anxiety disorders. Neurotherapeutics.

[30] Merrick J (2016). Identification of psychoactive degradants of cannabidiol in simulated gastric and physiological fluid. Cannabis Cannabinoid Res..

[31] Watanabe K (2007). Conversion of cannabidiol to 9-tetrahydrocannabinol and related cannabinoids in artificial gastric juice, and their pharmacological effects in mice. Forensic Toxicol..

[32] Breuer, A. *et al*. Fluorinated cannabidiol derivatives: Enhancement of activity in mice models predictive of anxiolytic, antidepressant and antipsychotic effects (vol 11, e0158779, 2016). *PloS One* **11**, 10.1371/journal.pone.0162087 (2016). 10.1371/journal.pone.0158779 PMC4945002 27416026

[33] Kano M, Ohno-Shosaku T, Hashimotodani Y, Uchigashima M, Watanabe M (2009). Endocannabinoid-mediated control of synaptic transmission. Physiol. Rev..

[34] Citraro R (2013). Cb1 agonists, locally applied to the cortico-thalamic circuit of rats with genetic absence epilepsy, reduce epileptic manifestations. Epilepsy Res..

[35] Deshpande LS (2007). Cannabinoid cb1 receptor antagonists cause status epilepticus-like activity in the hippocampal neuronal culture model of acquired epilepsy. Neurosci. Lett..

[36] Crombie L, Crombie WML, Firth DF (1988). Terpenylations using (R)-(−)–phellandrene. synthesis of the (3S,4R)-8,9-dihydro-o-and-p-cannabidiols, their iso-thc’s, and the natural dihydrochalcone (3S,4R)-(+)-linderatin. J. Chem. Soc., Perkin Trans..

[37] Bardyshev I, Barkhash V, Dubovenko Z, Lysenkov V (1978). Generation of a stable allyl cation from p-mentha-1,8-diene. Zh. Org. Khim..

[38] Focella A, Teitel S, Brossi A (1977). A simple and practical synthesis of olivetol. J. Org. Chem..

[39] Jones NA (2010). Cannabidiol displays antiepileptiform and antiseizure properties *in vitro* and *in vivo*. J. Pharmacol. Exp. Ther..

[40] Leite R, Carlini EA, Lander N, Mechoulam R (1982). Anticonvulsant effects of the (−) and (+) isomers of cannabidiol and their dimethylheptyl homologs. Pharmacology.

[41] Grotenhermen F, Muller-Vahl K (2016). Medicinal uses of marijuana and cannabinoids. Crit. Rev. Plant Sci..

[42] McGrath JC, Drummond GB, McLachlan EM, Kilkenny C, Wainwright CL (2010). Guidelines for reporting experiments involving animals: the arrive guidelines. Br. J. Pharmacol..

[43] Kilkenny C, Browne WJ, Cuthill IC, Emerson M, Altman DG (2010). Improving bioscience research reporting: The arrive guidelines for reporting animal research. J. Pharmacol. Pharmacother..

[44] Zgair A (2015). Development of a simple and sensitive hplc-uv method for the simultaneous determination of cannabidiol and delta(9)-tetrahydrocannabinol in rat plasma. J. Pharm. Biomed. Anal..

